# Public Knowledge, Awareness, and Vaccination Rates for Hepatitis B in India: A Cross-Sectional Survey

**DOI:** 10.7759/cureus.43997

**Published:** 2023-08-23

**Authors:** Ashish Kumar, Anil Arora, Praveen Sharma, Naresh Bansal, Shrihari A Anikhindi, Shivam Khare, Mandhir Kumar, Piyush Ranjan, Munish Sachdeva

**Affiliations:** 1 Institute of Liver, Gastroenterology, & Pancreatico-Biliary Sciences, Sir Ganga Ram Hospital, New Delhi, IND

**Keywords:** demographics, awareness, cross-sectional study, public health, online survey, general public, vaccination status, knowledge, liver disease, hepatitis b

## Abstract

Background: Hepatitis B virus (HBV) is a considerable public health concern in India, characterized by a significant number of chronically infected individuals. India falls into the category of an intermediate HBV endemic country, with approximately 40 million people chronically infected. Public awareness and knowledge about HBV are crucial for prevention, diagnosis, and treatment. This study aimed to assess the knowledge and vaccination status of the general public regarding hepatitis B in India.

Methods: An online cross-sectional survey was conducted with 3,545 participants, excluding healthcare workers and individuals below 18 years old. The survey evaluated knowledge in four domains: cause and spread of the disease (Domain A), organ affected and consequences (Domain B), treatment (Domain C), and vaccination (Domain D). The survey used a scoring scale from -20 to +22 points. Vaccination status was also assessed.

Results: The mean knowledge score of the respondents was 6.89 (±5.88). Only 25% of the respondents demonstrated good knowledge (score ≥12), while 31% had fair knowledge (score 6-11), 40% had poor knowledge (score 0-5), and 4% had incorrect knowledge (score <0). Males, individuals aged 18-60 years, respondents with higher education levels, and urban residents had significantly higher mean knowledge scores (p < 0.01). In the domain-specific analysis, 47% of the respondents had good knowledge in Domain B, while 43% of the respondents had good knowledge in Domains C and D. Only 17% of the respondents had good knowledge in Domain A. Overall, 22.7% of the respondents had received all three doses of the HBV vaccine, with higher vaccination rates among males, individuals aged 18-60 years, those with higher education levels, and urban residents.

Conclusion: The study revealed limited knowledge about HBV among the general public in India. Vaccination rates were also suboptimal. Public health interventions, including educational campaigns and targeted vaccination programs, are needed to improve awareness and vaccination coverage, ultimately reducing the burden of HBV infection.

## Introduction

Hepatitis B virus (HBV) infection remains a significant global public health concern, with an estimated 296 million people living with chronic HBV infection and nearly 887,000 deaths occurring annually due to its complications, such as cirrhosis and hepatocellular carcinoma [1-4]. Despite the availability of an effective vaccine for more than three decades, the incidence of HBV infection remains high, particularly in low- and middle-sociodemographic index (SDI) countries, where the majority of HBV infections occur [5].

There is a lack of large-scale population studies on the prevalence of HBV in India, with most data coming from blood bank screening. India is classified as an intermediate HBV endemic country, with an estimated 40 million chronically infected individuals [6]. Estimates of HBV prevalence vary widely, with the overall rate of hepatitis B surface antigen (HBsAg) positivity ranging from 2% to 8%, but a recent meta-analysis suggests an overall prevalence of 3.70% (corresponding to a chronic carrier rate of 2.96%), indicating that India might have more than 37 million HBV carriers [7]. Geographic variations in prevalence may be due to differences in socioeconomic status or cultural practices [5].

Several studies have been conducted globally to assess the knowledge, awareness, and vaccination status of hepatitis B among various populations [8-22]. However, there is limited data on the knowledge and vaccination status of the general public in India. Understanding the knowledge gaps and vaccination coverage is crucial for designing and implementing public health interventions to reduce the burden of HBV infection in the country.

This study aimed to assess the knowledge and vaccination status of hepatitis B among the general public in India using an online cross-sectional survey. The findings of this study will provide valuable insights for policymakers and healthcare providers to improve public awareness, increase vaccination coverage, and ultimately reduce the burden of hepatitis B in India.

## Materials and methods

Study design and participants

A cross-sectional survey was conducted to assess the knowledge and vaccination status of hepatitis B among the general public in India. The study population comprised adults aged 18 years and above, excluding healthcare workers.

Aims and objectives

The two specific objectives of the study were (1) to assess the knowledge of the general public regarding hepatitis B in India, specifically focusing on understanding the cause and spread of the disease, the affected organ and its consequences, treatment options, and vaccination, and (2) to evaluate the vaccination status of the general public in relation to hepatitis B in India, including the proportion of respondents who had received all three doses of the HBV vaccine and identifying any demographic factors associated with higher vaccination rates.

Participants

The inclusion criteria were all consenting adults aged 18 years and above, while the exclusion criteria were healthcare workers.

Data collection

The survey was conducted using a validated questionnaire, which was administered through the SurveyMonkey platform. The questionnaire was distributed to potential participants via text message, social media, and WhatsApp. The participants provided informed consent before responding to the survey. The institutional ethics committee of Sir Ganga Ram Hospital, New Delhi, India, approved the study on August 9, 2021 (vide EC/07/21/1920). Repeated participation by the same person (using the same IP address) was prevented by the survey platform.

The questionnaire was carefully designed to cover various aspects of hepatitis B knowledge and awareness among the general public, providing a comprehensive understanding of the participants' knowledge and vaccination status related to hepatitis B. To ensure the validity of our scoring system, we undertook a thorough process of questionnaire validation and piloting, which was carried out in a test sample of 635 subjects. During this process, the consistency and reliability of the questionnaire (including the scoring criteria) was assessed. The validation phase aimed to confirm that the scoring system appropriately differentiates between participants' knowledge levels and aligns with established standards for evaluating health-related knowledge in public health research. The validation was done by the "Validation Committee" of Sir Ganga Ram Hospital, and the final approval was given on December 14, 2021. The internal consistency of the questionnaire, as measured by Cronbach's alpha, was found to be 0.799, indicating good reliability.

Survey questionnaire

The questionnaire used for this survey was designed by the authors to assess the knowledge and awareness of hepatitis B among the general public in India. The design process involved an extensive review of existing literature [8-22] on HBV awareness and knowledge. While the questionnaire was not directly modeled after any specific existing surveys, the domains were chosen based on their recurring importance in the existing scientific literature on HBV awareness. The aim was to create a set of questions that would adequately cover public knowledge in four distinct domains.

The questionnaire was divided into several sections, comprising a total of 22 questions (Table 1).

**Table 1:** Hepatitis B (HBV) knowledge questionnaire and the point criteria. Maximum points 22, minimum points -20. Good knowledge ≥12 points; fair knowledge = 6-11 points; poor knowledge = 0-5 points; wrong knowledge <0 points

Domains	#	Question	Response	Points awarded on clicking
Domain A: Knowledge about cause of disease and its method of spread (Max 12, min -10) Good: 7 – 12 Fair: 4 – 6 Poor: 0 – 3 Wrong: <0	1	"Hepatitis B" disease is caused by? (Single response) Max 1, min -1	It is caused by a bacteria.	-1
			It is caused by a virus.	1
			Don't know / blank	0
	2	What are the modes of spread of "Hepatitis B"? (Multiple response) Max 7, min -5	Unsterilized needle	1
			Sharing razor	1
			Shaking hands	-1
			Mother to baby	1
			Infected food	-1
			Coughing without mask	-1
			Unprotected sex	1
			Tattooing	1
			Blood transfusion	1
			Hugging	-1
			Sharing needles of IV drugs	1
			Donating blood	-1
	3	Is "Hepatitis B" more infectious than HIV or AIDS? (Single response) Max 1, min -1	Yes	1
			No	-1
			Don't know / blank	0
	4	If you have undergone surgery/dental treatment/blood transfusion/tattooing in the past, should you be tested for "Hepatitis B"? (Single response) Max 1, min -1	Yes	1
			No	-1
			Don't know / blank	0
	5	If someone has "Hepatitis B," should all household members be tested for "Hepatitis B"? (Single response) Max 1, min -1	Yes	1
			No	-1
			Don't know / blank	0
	6	Should pregnant woman be tested for "Hepatitis B"? (Single response) Max 1, min -1	Yes	1
			No	-1
			Don't know / blank	0
Domain B: Knowledge about which organ gets affected by HBV and what HBV can cause (Max 4, min -4) Good: 3 – 4 Fair: 2 Poor: 0 – 1 Wrong: <0	1	"Hepatitis B" is known to damage which organ of the body the most? (Single response) Max 1, min -1	Lungs	-1
			Liver	1
			Kidney	-1
			Heart	-1
			Don't know / blank	0
	2	Can "Hepatitis B" cause jaundice? (Single response) Max 1, min -1	Yes	1
			No	-1
			Don't know / blank	0
	3	Can "Hepatitis B" cause cancer of liver? (Single response) Max 1, min -1	Yes	1
			No	-1
			Not sure / blank	0
	4	If a patient of "Hepatitis B" also has obesity or diabetes, will he have more severe disease? (Single response) Max 1, min -1	Yes	1
			No	-1
			Don't know / blank	0
Domain C: Knowledge about treatment (Max 3, min -3) Good: 2 – 3 Fair: 1 Poor: 0 Wrong: <0	1	Is "Hepatitis B" treatable by medicines? (Single response) Max 1, min -1	Yes	1
			No	-1
			Don't know / blank	0
	2	Should a patient suffering from "Hepatitis B" need to be isolated? (Single response) Max 1, min -1	Yes	1
			No	-1
			Don't know / blank	0
	3	Can alcohol cause more damage to the liver if the patient is infected by "Hepatitis B"? (Single response) Max 1, min -1	Yes	1
			No	-1
			Don't know / blank	0
Domain D: Knowledge about vaccination (Max 3, min -3) Good: 2 – 3 Fair: 1 Poor: 0 Wrong: <0	1	Are safe vaccines available for prevention of "Hepatitis B"? (Single response) Max 1, min -1	Yes	1
			No	-1
			Don't know / blank	0
	2	Who should receive "Hepatitis B" vaccine? (Single response) Max 1, min -1	Children only	-1
			Adults only	-1
			Both children and adults	1
			Don't know	0
	3	For how many years will the immunity against "Hepatitis B" last if "Hepatitis B" vaccine has been administered in proper dose and schedule? (Single response) Max 1, min -1	One year	-1
			5 years	-1
			Lifelong	1
			Don't know	0

The domains and their respective questions are as follows: (1) Inclusion and exclusion criteria (two questions): These questions were used to determine eligibility for participation in the survey based on the predefined inclusion and exclusion criteria. (2) Demographic profile of subjects (four questions): This section collected demographic information of the participants, including age, gender, education level, and geographic location. (3) Domain A (knowledge about the cause of disease and its method of spread (six questions)): This domain assessed participants' understanding of the causes of hepatitis B and how the virus is transmitted from one person to another. (4) Domain B (knowledge about which organ gets affected by HBV and what HBV can cause (four questions)): This section evaluated the participants' knowledge of the primary organ affected by hepatitis B (the liver) and the potential complications or consequences of the disease, such as cirrhosis, liver failure, and liver cancer. (5) Domain C (knowledge about treatment (three questions)): This domain assessed the participants' awareness of the available treatment options for hepatitis B, including antiviral medications and liver transplantation, and the importance of regular monitoring and follow-up care. (6) Domain D (knowledge about vaccination (three questions)): This section evaluated the participants' understanding of the hepatitis B vaccine, its effectiveness, and the recommended vaccination schedule. (7) Vaccination status of the subject (one question): This question aimed to determine the vaccination status of each participant, specifically whether they had received the hepatitis B vaccine or not.

Scoring of points in the questionnaire

The scoring system for the questionnaire was designed to assess the participants' knowledge about hepatitis B based on their responses. The questionnaire had a total of 22 questions, with a maximum possible score of 22 points and a minimum possible score of -20 points. The scoring categories were as follows: (A) Good knowledge (≥12 points): Participants who scored 12 points or higher were considered to have a good knowledge of hepatitis B. This indicates a strong understanding of the disease, its causes, transmission, affected organs, complications, treatment options, and vaccination. (B) Fair knowledge (6-11 points): Participants who scored between 6 and 11 points were considered to have fair knowledge about hepatitis B. This suggests a moderate understanding of the disease and its related aspects, but it also indicates that there might be room for improvement in certain areas. (C) Poor knowledge (0-5 points): Participants who scored between 0 and 5 points were considered to have poor knowledge of hepatitis B. This indicates a limited understanding of the disease, with significant gaps in knowledge about its causes, transmission, complications, treatment, and vaccination. (D) Wrong knowledge (<0 points): Participants who scored less than 0 points were considered to have wrong knowledge about hepatitis B. This suggests that not only do they lack accurate information about the disease, but they also hold misconceptions or incorrect beliefs about it.

The scoring system enabled the researchers to categorize the participants' knowledge about hepatitis B effectively, helping to identify areas where educational interventions may be needed to improve public understanding of the disease.

Data analysis

Descriptive statistics were used to summarize the demographic characteristics of the participants and their knowledge and vaccination status concerning hepatitis B. Frequencies and percentages were calculated for categorical variables, while means and standard deviations were computed for continuous variables. The internal consistency of the questionnaire was assessed using Cronbach's alpha to determine the reliability of the items. Associations between demographic factors, knowledge, and vaccination status were examined using appropriate statistical tests. Statistical analysis was performed using IBM SPSS Statistics version 25 (released 2017; IBM Corp., Armonk, NY, USA). The significance level was set at p < 0.05.

## Results

Demographic profile of the respondents

The survey was rolled out on December 15, 2021, and the last response was recorded on March 11, 2023. During this period, the total number of responses received was 4,158. Of these, healthcare workers were 507 and subjects below 18 years of age were 106, so these responses were excluded. Hence, the remaining 3,545 responses were included for analysis.

Table 2 provides information on the demographics of respondents.

**Table 2:** Demographic profile of the included respondents.

Parameter	Characteristics	N = 3545
Age in years	18-60	3167 (89%)
	>60	316 (9%)
	Did not reveal	62 (2%)
Gender	Males	2167 (61%)
	Females	773 (22%)
	Did not reveal	605 (17%)
Education status	Graduate and post-graduate level	2458 (69%)
	Educated till 12th class	320 (9%)
	Less than 12th class	170 (5%)
	Did not reveal	597 (17%)
Residence	Urban	2574 (73%)
	Rural	381 (11%)
	Did not reveal	590 (17%)

The majority of the participants were between 18 and 60 years old, with 89% falling within this age range, while only 9% were over 60 years old and 2% did not reveal their age. In terms of gender, 61% were males, 22% were females, and 17% did not reveal their gender. Regarding education status, 69% had graduated or completed post-graduate education, 9% had completed education till 12th class, and 5% had education less than 12th class. A proportion (17%) did not reveal their education status. Finally, 73% of the participants lived in urban areas, 11% in rural areas, and 17% did not reveal their place of residence.

Overall score of the respondents

The mean score obtained by all the respondents (n = 3545) was 6.89 (±5.88). The maximum score was 22 and the minimum score was -10. Only 882 (25%) respondents had good knowledge and scored ≥12 points, while 1111 (31%) respondents had fair knowledge and scored between six and 11 points; 1407 (40%) had poor knowledge and scored between zero and five points, and 145 (4%) respondents had wrong knowledge and scored less than zero points.

**Figure 1:**
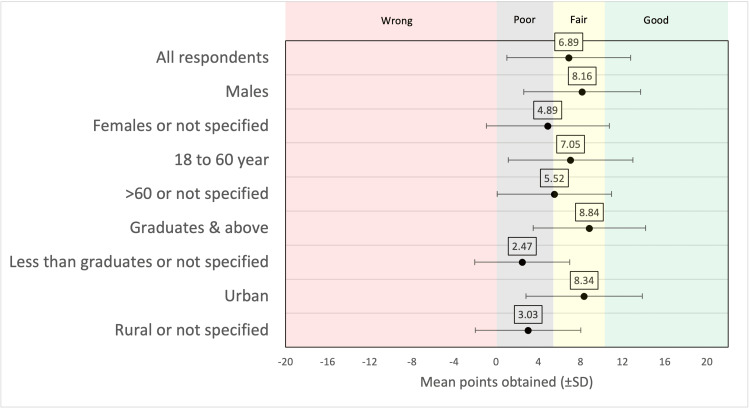
Forest plot of the means scores of various subgroups based on the demographic characteristics of the respondents.

Figure 1 shows the forest plot of the means scores of various subgroups based on the demographic characteristics of the respondents.

Males scored higher points (mean 8.16 ± 5.54) than females and those who did not specify their gender (4.89 ± 5.84; p < 0.01). The respondents in the age group of 18 to 60 years scored higher points than those who are more than 60 year old or those who did not specify (7.05 ± 5.91 vs. 5.52 ± 5.44; p < 0.01). The respondents who were graduates or attained post-graduate education scored higher points than those who were less educated or those who did not specify (8.84 ± 5.33 vs. 2.47 ± 4.52; p < 0.01). Finally, respondents living in the urban area scored more points than those living in the rural area or those who did not specify (8.34 ± 5.51 vs. 3.03 ± 5.01; p < 0.01).

Domain-specific analysis

The survey questionnaire tested the respondents’ knowledge on HBV in four domains: Domain A (knowledge about cause of disease and its method of spread), Domain B (knowledge about which organ gets affected by HBV and what HBV can cause), Domain C (knowledge about treatment), and Domain D (knowledge about vaccination).

**Figure 2:**
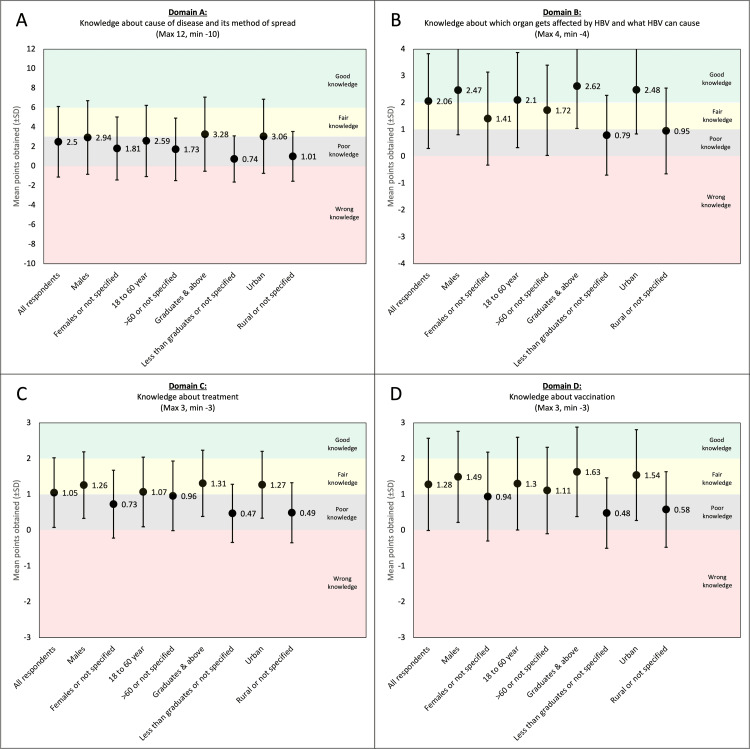
Domain-wise mean score of all respondents and the mean scores of various demographic subgroups.

Figure 2 shows the domain-wise mean score of all respondents and the mean scores of various demographic subgroups.

In Domain A, the maximum possible score was 12 and the minimum was -10. The mean score of all the respondents was 2.5 ± 3.62 points. The mean scores of all the subgroups were below 3, except the subgroups of graduate and post-graduate level respondents and urban respondents, both of which had scores barely above 3. In Domain B, the maximum possible score was 4 and the minimum was -4. The mean score of all the respondents was 2.06 ± 1.77. In the subgroup analysis, the mean scores of the male respondents, 18- to 60-year-old respondents, graduate and post-graduate level respondents, and urban respondents were above 2. In Domain C, the maximum possible score was 3 and the minimum was -3. The mean score of all the respondents was 1.05 ± 0.97. The mean scores of the male respondents, 18- to 60-year-old respondents, graduate and post-graduate level respondents, and urban respondents were above 1. Similarly, in Domain D, the maximum possible score was 3 and the minimum was -3. The mean score of all the respondents was 1.28 ± 1.29. The mean scores of the male respondents, 18- to 60-year-old respondents, more than 60-year-old respondents, graduate and post-graduate level respondents, and urban respondents were above 1.

**Figure 3:**
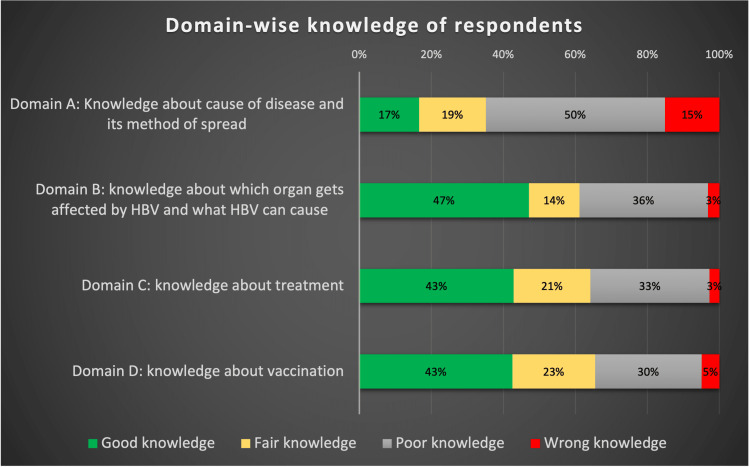
Domain-wise knowledge of respondents categorised into good knowledge, fair knowledge, poor knowledge and wrong knowledge. Domain A: Maximum points 12, minimum points -10; good knowledge: 7 to 12 points; fair knowledge: 4 to 6 points; poor knowledge: 0 to 3 points; and wrong knowledge: <0 points. Domain B: Maximum points 4, minimum points -4; good knowledge: 3 to 4 points; fair knowledge: 2 points; poor knowledge: 0 to 1 points; and wrong knowledge: <0 points. Domain C: Maximum points 3, minimum points -3; good knowledge: 2 to 3 points; fair knowledge: 1 point; poor knowledge: 0 points; and wrong knowledge: <0 points. Domain D: Maximum points 3, minimum points -3; good knowledge: 2 to 3 points; fair knowledge: 1 point; poor knowledge: 0 points; and wrong knowledge: <0 points.

When the scores obtained in each domain were categorized into good knowledge, fair knowledge, poor knowledge, and wrong knowledge, it was observed that 17% respondents had good knowledge in Domain A, 47% had good knowledge in Domain B, and 43% had good knowledge in Domains C and D (Figure 3).

Vaccination status of the respondents

On enquiring about the HBV vaccination status of respondents, it was found that only 22.7% of the respondents had received all three doses of the HBV vaccine, 8.6% were inadequately vaccinated, 18.5% were unvaccinated, and the remaining 50.3% of the respondents were not sure whether they were vaccinated or not (Table 3).

**Table 3:** Vaccination status of the respondents.

Group	Fully vaccinated	Inadequate vaccinated	Not vaccinated	Unaware	P value
All respondents	803 (22.7%)	304 (8.6%)	655 (18.5%)	1783 (50.3%)	–
18-60 years old	739 (23.3%)	282 (8.9%)	572 (18.1%)	1574 (49.7%)	0.002061
>60 years old or did not reveal	64 (16.9%)	22 (5.8%)	83 (22.0%)	209 (55.3%)	
Males	542 (25.0%)	223 (10.3%)	484 (22.3%)	918 (42.4%)	<0.00001
Females or did not reveal	261 (18.9%)	81 (5.9%)	171 (12.4%)	865 (62.8%)	
Graduate or post-graduate level	700 (28.5%)	258 (10.5%)	567 (23.1%)	933 (38%)	<0.00001
Less than graduate level or did not reveal	103 (9.5%)	46 (4.2%)	88 (8.1%)	850 (78.2%)	
Urban	713 (27.7%)	274 (10.6%)	563 (21.9%)	1024 (39.8%)	<0.00001
Rural or did not reveal	90 (9.3%)	30 (3.1%)	92 (9.5%)	759 (78.2%)	

In the subgroup analysis, according to the demographic categories, in terms of age, 23.3% of the subjects in the 18-60-year-old group are fully vaccinated for HBV, while 16.9% of the >60-year-old group or those who did not reveal their age are fully vaccinated. When it comes to gender, 25% of the male subjects are fully vaccinated for HBV, while only 18.9% of the female subjects or those who did not reveal their gender are fully vaccinated. Regarding education status, 28.5% of the subjects who are graduates or hold a higher degree are fully vaccinated for HBV, while only 9.5% of those with education below graduate level or those who did not reveal their educational background are fully vaccinated. Finally, with respect to residence, 27.7% of the subjects living in urban areas are fully vaccinated for HBV, while only 9.3% of those residing in rural areas or those who did not reveal their place of residence are fully vaccinated.

## Discussion

Our survey results show that the Indian general public's knowledge of HBV is suboptimal, with only 25% of the respondents demonstrating good knowledge (≥12 points) and 44% having either poor or incorrect knowledge. Our survey has several strengths that lend credibility to its findings. First, the large sample size of 3,545 respondents increases the statistical power and generalizability of our results. Second, the comprehensive survey design, which assessed knowledge across four domains (cause of disease and method of spread, affected organ and consequences, treatment, and vaccination), allows for a more in-depth understanding of the respondents' knowledge gaps and areas of strength. In addition, the inclusion of demographic variables (age, gender, education, and residence) enabled us to identify specific subgroups with lower knowledge scores and vaccination rates, providing valuable insights for targeted interventions. Lastly, the extended data collection period, spanning from December 2021 to March 2023, offers a more accurate representation of the general public's knowledge and vaccination status over time, accounting for potential fluctuations due to changes in public health messaging or other external factors.

This study sheds light on the public's understanding and hepatitis B vaccination status in India, revealing an overall suboptimal level of knowledge, with just 25% of participants demonstrating a good level of understanding [8-11]. This deficit mirrors findings from other global regions and underpins a concerning gap in public awareness around HBV's transmission methods, potential complications, treatment options, and preventative measures, all of which are paramount for the virus's control and mitigating its public health impact [3].

Our findings align with previous studies showcasing similar subpar levels of hepatitis B awareness across different populations and geographical locations [11-22]. This further emphasizes the need for robust educational interventions to enhance public understanding of hepatitis B.

Our survey unveiled trends within the dataset, identifying males, younger respondents (18-60 years), those with higher education levels, and urban residents as having relatively higher knowledge scores across all domains. These demographic and socioeconomic factors seem to influence the level of knowledge about hepatitis B, an insight that aligns with previous studies and could guide future targeted educational campaigns [23]. Conversely, we identified females, individuals over 60 years, those with lower education levels, and rural dwellers as groups with lower knowledge scores, corroborating findings from other studies [11-14,16,18]. This disparity underscores the need for tailored educational interventions for these subgroups to bridge knowledge gaps and improve overall hepatitis B awareness. Consequently, it appears paramount for public health initiatives in India to prioritize targeted education, particularly among women, the elderly, individuals with lower education levels, and those in rural areas to enhance hepatitis B literacy and improve public health outcomes.

The domain-specific knowledge analysis exposed distinct areas of understanding among respondents. Only 17% had a good grasp of the disease's cause and its transmission methods, indicating a considerable gap in comprehension that could contribute to ongoing virus transmission [24]. This area, labeled as Domain A in our study, pinpointed the lowest mean score among all categories and thus necessitates particular emphasis in future educational interventions. Contrastingly, the respondents showcased higher knowledge scores in domains related to the affected organ, potential health outcomes, treatment, and vaccination. Notably, Domain B, focusing on the organ affected by HBV and the potential consequences of the infection, yielded the highest mean score. This might be due to the widespread awareness that the liver is the primary organ impacted by hepatitis B, thereby indicating an existing foundation upon which to build further public understanding.

Regarding vaccination status, the study found a concerning shortfall with only 22.7% of respondents having received all three doses of the HBV vaccine, falling significantly short of the World Health Organization's target of 90% coverage [25]. These low rates mirror findings from previous studies underscoring similar issues in India [11,13,26]. Certain subgroups, such as those with lower education levels or living in rural areas, demonstrated even lower vaccination rates [11,13,14]. The results further revealed that 18.5% of respondents remained unvaccinated, and 50.3% were unsure of their vaccination status, illuminating a significant public health risk given the serious complications of hepatitis B, including cirrhosis and liver cancer. Males, younger individuals, those with higher education, and urban residents exhibited higher vaccination rates, aligning with past studies. These observations underscore the urgent need for enhanced public health efforts, focusing not only on improving HBV knowledge but also on better accessibility to vaccination services, particularly for vulnerable groups. Further research is needed to devise the most effective strategies to raise awareness and boost vaccination uptake among these populations.

The limitations of our study include the reliance on self-reported data, which may be subject to recall bias, and the use of an online survey, which may not be representative of the general population due to potential differences in internet access or digital literacy. Nonetheless, our findings provide valuable insights into the current state of hepatitis B knowledge and vaccination status among the general public and highlight areas for future intervention.

The low level of knowledge about hepatitis B and the suboptimal vaccination rate among our survey respondents underscore the need for targeted public health interventions to improve awareness and vaccination coverage. Educational campaigns should focus on the general public, with particular emphasis on females, older individuals, those with lower education levels, and rural residents, who demonstrated lower knowledge scores and vaccination rates in our survey. In addition, efforts should be made to increase awareness about the importance of completing the full vaccination schedule, as partial vaccination was reported by 8.6% of our respondents. Comprehensive strategies addressing both health literacy and vaccination coverage are vital for achieving the national and global targets for HBV control [27].

## Conclusions

This study sheds light on the inadequate knowledge and low vaccination rates related to hepatitis B among the general public in India. The findings highlight that only a quarter of respondents demonstrated good knowledge, while a considerable proportion lacked accurate understanding. Similarly, the study identified a concerning shortfall in vaccination rates, with only 22.7% having completed the recommended vaccination schedule.

These outcomes underscore the urgency for targeted public health interventions. Tailored educational efforts are crucial, particularly for vulnerable groups, such as women, the elderly, those with lower education levels, and rural residents. Equally important is the need to enhance accessibility to vaccination services and promote adherence to the complete vaccination regimen. The study underscores the need for comprehensive initiatives that bridge knowledge gaps and enhance vaccination coverage. By addressing these issues, policymakers and healthcare providers can work toward reducing the impact of hepatitis B in India.
